# Selections and Abstracts

**Published:** 1897-10

**Authors:** 


### SELECTIONS AND ABSTRACTS.

### The Physician upon the Witness Stand.

A late New York *Medical Journal* contains an able paper on this important and interesting subject by Dr. W. L. Munro, of Providence, Rhode Island, and as this is of particular interest at present to our Georgia readers, we give part of the paper here:

Theoretically, the position of the medical expert upon the witness stand should be a proud and dignified one. His selection to perform such a duty should imply a deserved tribute to his intelligence, education, and experience.

Practically, the expert has fallen in honor, until, from being the trusted interpreter of science for the benefit of the judge and jury, he has become the paid partizan of one or the other side, selected for his willingness to lend his aid in support of the particular views which that side wishes to establish. He is even to be congratulated if he does not find himself sunken still lower and simply a shuttlecock between the rackets of opposing counsel, each of whom is striving to use him and his *confreres*, not as a means of eliciting truth, but with the plain intent of hopelessly confusing the jury upon the issues involved.

In a recent case of much note, in which so much and such conflicting medical evidence was introduced that it was practically thrown out by the jury when they came to consider the case, an expert, just released after a prolonged and skillful cross-examination, said to the cross-examining lawyer, "You did your best to confuse me." "Oh, no, doctor," was the bland reply, "I have no intention of confusing you; it's the jury I wish to confuse."

The term "witness" as applied to the medical expert, is a misnomer. It only rarely happens that the physician has any personal knowledge of the case in which he is called as an expert.

To return to the subject of which I was speaking: the expert is not properly a witness. He is called into court because his education, training, and experience fit him to act as the sworn interpreter to the judge and jury, of the facts and theories of medicine, or other sciences or arts, as it may happen, such matters being beyond the comprehension of those uneducated in that particular specialty.

Wharton says: "The distinction between expert witnesses and ordinary witnesses is this: the non-expert witness testifies to conclusions which may be verified by the adjudicating tribunal; the expert to conclusions which can not be so verified. The non-expert gives the results of a process of reasoning familiar to everyday life; the expert gives the results of a process of reasoning which can be determined only by special scientists."

The position of the expert is thus, in theory if not in practice, quasi-judicial; he is the *amicus curiæ* of the law. Our judges and lawyers are slow to recognize and acknowledge this fact.

In Germany and France the medical expert still retains his position as one of the appointed officers of the court, a recognized part of the judicial system, and, as such, enjoys honor and his opinions are accepted as authoritative. His statement is final; he is not confronted with opposing experts; he is not subject to cross-examination. He is paid by the court and continues to serve for years.

This is the ideal, but, unfortunately, it is not in harmony with nor acceptable to the judicial systems of England and America, which show very great regard for the rights and privileges of individuals, and very little regard for absolute authority, of whatever sort, except the obsolete traditions by which the legal profession is too often guided.

In the latter part of the eighteenth century we first find reference made to paid partizan experts. There is no question so poor that it has not two sides. Hence, the moment an expert was called in by one party an opposing expert was summoned by the other side, and thus began the fall from grace which has robbed the expert of nearly or quite all of his old-time honor, and, only too often, has made him a laughing-stock for the public and converted so-called judicial proceedings into a farce to be settled one way or the other, according to the length of the purses of the respective litigants.

The late Dr. Wilber, of Syracuse, wrote: "Expert testimony should be the colorless light of science brought to bear upon any case where it is summoned. It should be impartial, unprejudiced. There should be no half-truths uttered, and suppression of the whole truth is in the nature of false testimony."

No nobler standard for the expert could possibly be established, and there have been and are many expert witnesses whose constant endeavor it is to live up to it. Such are deservedly honored and sought after. That they are in a hopeless minority will be shown to be the fault of the system which employs him rather than of the individual expert. The result, however, is the same wherever the blame is to be assigned, and the abuse long ago assumed startling proportions.

Admitting that medical expert testimony no longer receives the respect nor carries the weight of authority that it once did, let us consider to what its decline is attributable.

At the outset, the lack of any proper qualifications as to the competency and honesty of the proposed expert must be held responsible. "The courts accept any one as an expert and trust that cross-questioning will show that he is an ignoramus, if he really is such." Manifestly, the law has no right to recognize any one or other school of medicine. Science is ever advancing, and that which is all but universally subscribed to to-day may be forgotten or condemned to-morrow. Hence, every medical man of whatever school, or even of no school at all, must be accepted if brought forward by either side, provided that he proves upon examination to possess qualifications, which may vary widely according as the whim of the presiding judge or the exigencies of the case may dictate. This rule has even been extended, in certain cases, to admit nurses and medical students to expert standing. Practically, the fact that the would-be expert is a licensed practitioner carries with it presumptive evidence of his possessing a sufficient degree of knowledge and skill. The first few questions addressed to the expert are for the purpose of bringing out his qualifications. It is within the discretion of the opposing counsel to cross-examine the witness on this point if he sees fit. If counsel are disagreed, it becomes a matter for the court to decide. Where there is no contention the judge can not interfere, unless it happens that of his own personal knowledge he knows the expert in question to be unworthy of belief. This exceedingly loose method of testing the proposed witness, by sanctioning men of no standing or experience, does much to degrade the value of medical expert testimony.

Again, science itself is advancing with prodigious strides, and nowhere is this progress more marked than in medicine and surgery. It follows necessarily that there can be no certainty of agreement even between witnesses of ability and honesty, and this fact is often taken advantage of by counsel to throw undeserved ridicule on their conflicting theories. If, however, their opinions, and reasons therefor, are stated in plain terms, without ambiguity and without heat, above all with no suspicion of partizan bias, they will at least be heard with respect and go to the jury without discredit.

Moreover, the medical man himself is much to blame. Appreciation of the honor still supposed to pertain to the position of expert witness, a still keener appreciation of the possible fees to be gained, prior interest in the case, and a host of other motives, good and bad, lead to too great willingness to serve upon his part. Too often, as I have said, the medical expert goes into the case as a pronounced partizan, and grossly discredits the science of which he professes to be the exponent by his one-sided presentation of the points involved. He thus becomes a fair prey for the cross-examiner. Too often the medical expert, even if of undoubted ability, fails to appreciate the importance of carefully preparing himself for the ordeal, so as to do credit to himself and his calling. He thus exposes himself to attack and rout, and the opprobrium falling upon the profession is the greater in proportion as his standing in the community is the more assured.

By far the greatest responsibility for the decline of the expert lies with the system itself under which he serves. The expert witness is no longer, as a rule, called into the case to interpret to judge and jury matters of evidence, which are to them incomprehensible, but which his education and experience fit him to explain. He is summoned by one or the other party to the suit, either because he is known to incline toward their side of the controversy, or because it is believed that he can be brought to advocate the opinions which are to be proved. The expert thus enters into the case with a strong bias. With this bias it is but natural that he should embrace with alacrity whatever helps to establish his client's case, while he looks with a cold and critical eye upon anything which tends to weaken it. It is known, too, to the judge and jury that he has been retained to testify in favor of the party calling him. "Hence it is that, apart from the partizan temper, more or less common to experts, their utterances, now that they have as a class become the retained agents of the parties, have lost all judicial authority and are entitled only to the weight which a sound and cautious criticism would award to the testimony itself." (Woodson.)

The method of applying this "sound and cautious criticism" was thus summarized in a recent address by a distinguished corporation counsel:

"The expert gives his opinion and his reasons for it. An advocate, more or less learned in the law and superficially prepared for the purpose, with the aid of the opposing expert, puts the witness through a cross-examination, the object of which is not to learn the truth, but to demonstrate that his opinion is wrong, his reasons unsound, and the witness himself either ignorant, dishonest, or ridiculous. The expert on the other side is then called and gives a contrary opinion with grave and weighty reasons in support of it. The opposing lawyer then tries to show, by cross-examination, that this witness is as ridiculous, dishonest, ignorant, and unsound as his own expert. The whole matter is then referred to the jury, who were admittedly, at the outset, incapable of drawing a correct inference, but who, edified and instructed by the dignified performance which has taken place before them, are now expected to give the conflicting theories of the 'experts' the weight which 'a sound and cautious criticism' would award to them. The wisdom of such a final reference must be apparent; the capacity of the ordinary jury for 'sound and cautious criticism' in matters of science needs no eulogy here." (Mather's Address before the American Academy of Railway Surgeons in Chicago, September 25, 1896.)

Not infrequently the cross-examination of the experts develops into an undignified contest of wits—a wrangle, in fact, between witness and cross-examiner, in which the latter has every advantage from his knowledge of the courts and court procedure and the entire absence of embarrassment at being placed in an unaccustomed position. His very ignorance of the point under discussion stands him in good stead, as he absolutely fails to see the many scientific limitations which hedge about the expert in forming his opinion, and can count upon the jury, more ignorant than himself in matters of science, for sympathy in his effort to hold the medical expert up to ridicule. A powerful and most unjust weapon is placed in his hands by giving him the right to demand a categorical answer. The expert, familiar with his subject, realizes fully that such an answer is impossible. He gets but little comfort from the court, who, when appealed to, replies: "Answer Yes or No, and explain afterward, if you wish"; which amounts to saying "Commit yourself to an untruth if the counsel wishes it; you can deny or modify it afterward." As well say to the pugilist, "Allow yourself to be knocked down—you will be permitted to get up again." Thus we see how the judicial system itself, as applied to the experts, has been the most potent agent in their degradation.

While the medical profession has lost much of honor and dignity through the poor showing of its members upon the witness stand, it is not the only loser. Justice herself, and her servants, the legal profession, whose function it is now to support, now to subvert her, mourns the loss of the *amicus curiæ*, the trusted adviser, whose disinterested and faithful expositions of science did much to smooth her pathway.

Much thought has been bestowed upon the problem of how to restore the expert witness to some of his original influence and authority, and many theories have been proposed.

All schemes for the rehabilitation of the medical expert recognize the hopelessness of any reform which would retain the paid partizan witness, called by a side, and hence, as a rule, with a bias as hopeless as it is natural. It therefore happens that the various plans range all the way from the entire abolition of expert testimony to the appointment of an official expert, whose report shall be made in writing to the court and shall go to the jury with the stamp of official authority, no opportunity being allowed counsel for either side to insult his dignity or ruffle his composure by so disturbing a thing as a cross-examination. Without doubt the adoption of such a method would place the expert upon the highest possible plane and assist greatly toward the procurement of abstract justice; but it savors of despotism and is not in harmony with the genius of our institutions. Besides, abstract justice is very seldom what either side of a controversy desires to obtain.

In France the official expert can be examined, but the questions to be asked are determined beforehand by the court. Of the various modes of procedure in force in foreign countries the most practicable is that of Scotland, where the expert files a report upon which he may be cross-examined.

Within a few months this question was discussed by the able jurist whom I have before quoted.* His conclusions are of value as embodying most that is worth retaining in the many propositions. They are of special interest to us, as citizens of Rhode Island, from the many points of similarity between the remedies he advocates and the mode of procedure already established by our statutes.

After speaking of the present system of paid partizan experts, only to condemn it, he concludes:

"The remedy lies in the designation by the court, in each case, of the experts who shall be called upon to testify. Whether agreed upon by the parties or selected by the judge, the experts should receive their appointment from the court. They should be

*Mather.

officers of the court, sworn and acting as its commissioners for the better ascertainment of the truth." He would thus "insure the selection of actual experts," whose impartiality and disinterestedness could be counted upon, and hence, all partizanship being out of the question, the lawyers for each side would unite in desiring the selection of the expert from those most competent.

He "concedes the right of the expert to a reasonable or even liberal fee....Nor would he leave him, for the payment of his fee, to the hazard of the collection of a bill of costs. The payment of his fee should be required of the party demanding the appointment of the expert and as a condition precedent to the appointment." After stating that "logical adherence to the theory that the expert is the assistant and adviser of the court and jury, about matters of which both are ignorant, would require that he report his opinion with his reasons, orally or in writing, and that the jury should accept the opinion as conclusive of the question on which it bears," he regretfully admits that, since the opinions of the experts might differ, he "supposes that cross-examination must be admitted to play its part, though the moment the official expert submits to cross-examination he loses something of his dignity and independence as an agency and officer of the court."

Further, in civil suits arising out of personal injuries, "the injured person should be compelled to submit himself to a personal examination by the court's experts."

---

### THE TRUE TEST OF THE CRIMINAL RESPONSIBILITY OF THE INSANE.

There is a difference between an insane delusion which dominates and controls the action of an insane person, and a mere delusion which affects the sane or the insane mind similarly. Both sane and insane minds may rest under delusions, but whether the insane delusion be of such a character as to dominate the will and action of the accused, *in reference to the act*, is the crucial test of criminal responsibility.

No amount of moral degeneration or vice, which has become unresisted or irresistible, ever excuses crime. The second-nature criminal may have irresistible impulses to steal, rob, and commit crime. The light of science shines upon the path and clearly marks the boundary line of crime and vice in him who, dominated by an insane delusion, which controls the conduct and dominates the will, commits an act which lacks all the essential elements of crime. Chief Justice Gibson states the law correctly when he says in Commonwealth *vs.* Mosler, 4 Barr, 266: "It (insanity must amount to a delusion or hallucination controlling his will, and making the commission of the act a duty of overruling necessity," and again: "The law is that whether insanity be general or partial, it must be so great as to have controlled the will of its subject, and to have taken from him the freedom of moral action."

The knowledge of right and wrong, either in the abstract or in regard to the act committed, knowledge of its character and consequences, even, may exist, as in the case of Guiteau and possibly, though not probably, in the case of Dr. Beach (at the moment of the killing), then even, in the language of Judge Gibson, "If it (the insanity) was so great as to have controlled the will and taken from him the freedom of moral action," by the law of Pennsylvania the accused would not be responsible, and in cases of moral insanity under the law of that State, as announced by the court, Mr. Chief Justice Lewis pronouncing the opinion, "We say to you as the result of our reflections on this branch of the subject, that if the prisoner was actuated by an irresistible inclination to kill, and was utterly unable to control his will or subjugate his intellect, and was not actuated by anger, jealousy, revenge, and kindred evil passions, he is entitled to an acquittal."

The question is, or should be, How far does the delusion dominate the volition, or in another class of cases, as Sir James Stephen puts it, "Was the accused deprived, by a disease affecting the mind, of the power of passing a rational judgment on the moral character of the act which she meant to do?" (Bell's Medico-Legal Studies, vol. ii., p. 1.) Lord Denman said, in Rex *vs.* Oxford, 2 C. & P. 225, where the accused was evidently acting under the duress of a delusion probably of an insane character: "If some controlling disease was in truth the acting power within him which he could not resist, then he will not be responsible." In State *vs.* Pike, 49 N. H. 399 (50 N. H. 367).

The most complete recent statement of the law will be found in the opinion of Somerville, J., in Parsons *vs.* State, given in full in Bell's Med.-Legal Studies, vol. ii., p. 16. This holds that the injuries to be submitted to the jury in any criminal trial where the defence of insanity is interposed should be:

1. Was the defendant at the time of the commission of the alleged crime, as matter of fact, afflicted with a *disease of the brain affecting the mind*, so as to be either idiotic, or otherwise insane?

2. If such be the case, did he know right from wrong as applied to the particular act in question? If he did not have such knowledge, he is not legally responsible.

3. If he did have such knowledge, he may nevertheless not be legally responsible if the two following conditions occur:

(1) If, by reason of the duress of such mental diseases, he had so far lost the *power to choose* between the right and the wrong, and to avoid doing the act in question, as that his free agency was at the time destroyed.

(2) And if, at the same time, the alleged crime was so connected with such mental disease, in the relation of cause and effect, as to have been the product of it *solely*.

(Bell's Med.-Legal Studies, vol. ii., p. 31.) *Vide* also opinion by Dillon, C. J., in State *vs.* Felton, 35 Iowa, 67 (Bell's Med.-Legal Studies, vol. ii., p. 16). Judge Dillon held: That the capacity to distinguish between right and wrong was not a safe test of criminal responsibility in all cases, and it was accordingly decided, that, if a person commit a homicide, knowing it to be wrong, but do so under the influence of an uncontrollable and irresistible impulse, arising not from natural passion, but from an insane condition of the mind, he is not criminally responsible. "If," said Chief Justice Dillon, "by the observation and concurrent testimony of medical men who make the study of insanity a specialty, it shall be definitely established to be true that there is an unsound condition of the mind, that is, a diseased condition of the mind, in which, though a person abstractly knows that a given act is wrong, he is yet, by an *insane impulse*, that is, an impulse proceeding from a diseased intellect, irresistibly driven to commit it—the law must modify its ancient doctrines and recognize the truth, and give to this condition, when it is satisfactorily shown to exist, its exculpatory effect."

In the case of People *vs.* Daly—trial in 8th District Court at Washington, D. C., Jan. 13, 1897—Judge Montgomery charged the jury as follows:

1. Was the defendant at the time of the act, as matter of fact, afflicted with disease of the mind, was he wholly or partially insane?

2. If he *was* so afflicted, did he know right from wrong, as applied to the homicide in question?

If he *did* have such knowledge, had he by reason of the duress of such mental disease, so far lost the power to choose between the right and the wrong, and to avoid doing the act in question, as that his free agency was, at the time, destroyed, and if so, was the homicide so connected with such mental disease, in the relation of cause and effect, as to have been the product of it (the mental disease) solely. If you are satisfied from the evidence that the defendant was mentally afflicted, so that he did not know right from wrong, as applied to the act, or if he *did* know, but by reason of the duress, the stress of his mental disease (if he had any), he had no power to choose, no power to avoid doing what he did, and if the homicide was the product of his mental condition solely, or, if by reason of the insane delusions which the defendant had been harboring (if any), he had reached that condition of mind where the morbid impulse to kill became irresistible, and existed in such violence as to subjugate his intellect, control his will, and render it impossible for him to do otherwise than to yield and do as he *did*, then he is not to be held accountable.

"If some controlling (mental) disease was in truth the acting power within him, which he could not resist, then he will not be responsible."

"If a person commit a homicide under the influence of an unaccountable and irresistible impulse, arising *not* from natural passion, but from an insane condition of the mind, he is not criminally responsible."—[*From Advance Sheets Clark Bell's 12th American Edition Taylor's Medical Jurisprudence.*]

---

### RECENT FOREIGN LITERATURE ON APPENDICITIS.

The subject of appendicitis has been attracting anew the attention of physicians and surgeons on the continent of Europe. At the meeting of the German Society of Surgery, held April 21st, the importance of early surgical intervention was emphasized. This was also, in part, the consensus of the discussion before the Société de Médecine et de Chirurgie Pratique at a recent meeting. The French medical mind has in the past been inclined to conservatism, and it has been regarded as good practice to wait a week or longer—even in severe cases—in the hope that this perityphlitic abscess might be well walled in from the abdominal cavity.

Paul Reynier, at the above-mentioned meeting, advocated this procedure. In the immense majority of cases intervention should be tardy, and by this he meant, put off till some time between the fifth and ninth day. He would except appendicitis in young children, which has little tendency to be attended with neo-membranous formation and walling in of the suppuration. His experience with appendicitis in adults leads him to call especial attention to the proneness of this disease to be followed by a peritonitis which is distinctly localized. Out of forty cases he has seen but three deaths from general peritonitis. There is, therefore, a great gain in waiting till the peritonitis is localized in the cecal region, and adhesions are formed protecting the rest of the peritoneal cavity. In exceptional cases of the fulminant character, laparotomy should be performed at once, as soon as the diagnosis is made.

Dr. Louis Beurnier had little faith in the medical treatment of appendicitis. Every case of appendicitis is "of the province of the surgeon," and should be under the constant care and scrutiny of the surgeon, in readiness for an operation. In many cases it may be expedient to wait "till the acute period of the onset is passed, till the general condition is improved by injections of caffeine, and the local state is ameliorated by applications of ice," but the laparotomy should only in exceptional cases be deferred beyond the third or fourth day. He insists on the necessity of a free incision, prolonged well upward, so as to give plenty of room for exploration. If the pus does not flow by gentle maneuvers, use care in exploring with the finger so as not to break down adhesions. It is often better to leave the appendix *in situ.*

Dr. F. Verchère contributed to the same society a brilliant paper, in which he stated the view that there are two types of appendicitis, both having a different pathogeny and different clinical tableaux. There is no difficulty in accepting the two types; but is not the malignant form an aggravation of the simple benign type, due to the same microbes and toxins, only intensified by cause not yet well understood? We know that one type is relatively benign, characterized by a phlegmon of the right iliac fossa; this phlegmon may go on to suppuration, or it may remain "sero-fibrinous" and end in resolution. The sequels of the appendicitis may be those of an ordinary abscess opening externally or especially dangerous only if it bursts into the peritoneal cavity. Cases of this type do not show a marked degree of infection. The phagocytes of the individual affected early display their protective activity in a sufficient degree to get the upperhand of their bacterial adversaries, walling away from the abdominal cavity the seat of infection; and only a localized focus of suppuration attests the mischief wrought in the *vase close* of an occluded appendix.

The malignant type of appendicitis is appropriately called by Verchère "intestino-peritoneal septicemia." These cases are generally fatal, though a small percentage are saved by an early operation. Here there is intensification of the virus owing to favorable culture conditions in the "closed receptacle" and rapid absorption of toxins with generalization of the inflammation to the peritoneum and a speedy septicemic breakdown. There may be but little localization of the mischief, and from beginning to end no suppuration.

Paul Reclus writes on "The Pathogeny of Appendicitis." His paper deserves more notice than the limits of this article will allow. He shows that the *vase close* theory is more or less applicable to the disorder following all kinds of appendicitis; for when an inflammation is once started in that diverticulum, there is, owing to its peculiar shape and situation, the greatest tendency to stagnation of the contents, thus offering the best culture field to bacteria of the intestine, notably the streptococcus and coli bacillus. But many cases of appendicitis do not start in the appendix, but are propagated from an enteritis of the colon and cecum, and it has been of late proved that appendicitis sometimes originates from general causes, as infectious fevers. But whatever cause may have been operative, "the structure of the appendix, its canalicular form, its nature as a diverticulum, make it the equivalent of a blind natural fistula where microbes stagnate, and with exaltation of their virulence, invade its walls, cause ulceration and perforation, and easily by propinquity excite grave peritonitis."

The trend of the best surgical opinion, abroad as well as at home, appears to be in favor of early operation in the fulminating cases; while, in a very large class of cases, namely, those of more moderate severity, surgeons are learning to wait until the height of the attack is passed, when the abscess, if it forms, can be opened with comparative safety, and the appendix, if it can be reached without danger of rupturing adhesions and infecting the peritoneum, removed. After the patient has recovered from this operation, the appendix may, if not obliterated, and still causing trouble, be removed with comparative safety.

There is also a large class of cases in which an exudate, although comparatively large in an acute attack, is entirely absorbed by natural processes, and no further attack may follow, or the appendix may be removed by the comparatively safe internal operation. —*Editorial, Boston Med. and Surg. Journal.*

---

### CHRONIC GASTRITIS.

Prof. H. T. Webster, M.D., of San Francisco, in a recent publication, discusses chronic gastritis and its treatment. After a careful *resume* of the etiology and pathology of the affection, and an enumeration of the symptoms attending it, he says, in speaking of the diagnosis:

"The use of the stomach-tube will afford the best means of diagnosis. If siphonage be practiced an hour or so after eating, hydrochloric acid will usually be absent, and lactic acid, associated with fatty acids, is present with a large quantity of mucus. If siphonage be practiced seven hours after eating, undigested food will be found still remaining in the stomach, while in cases of functional dyspepsia it will have disappeared. Malignant disease will be excluded by lack of cachexia, absence of perceptible tumor upon palpation, and by the character of the material vomited, coffee ground material soon appearing in cancer. In gastric ulcer a diagnostic feature is frequent hematemesis."

He believes that if a proper diet be pursued and rational medicinal treatment be employed, almost every case of chronic gastritis will improve readily, unless it be complicated by gastric carcinoma, gastric ulcer, or hepatic, renal, or pulmonary disease. His treatment consists in lavage, disinfection and cleansing of the viscus with hydrozone. Lavage should be practiced every morning before eating, a small quantity of water (a pint) being used at first, which should be increased to two or three quarts as the treatment is carried on. The water should be warm (98.6 degrees F.) and solutions containing Glauber's salt, asepsin or boracic acid are often useful. Regarding the use of hydrozone in this affection, he says:

"The introduction of hydrozone as a remedy in this condition was an innovation of remarkable value. A drachm of Marchand's hydrozone, added to four ounces of boiled water, and drunk while the stomach is empty exerts a powerful influence in dissolving and removing the tenacious mucus, destroying microbic elements of fermentation and stimulating normal action in the diseased mucous surface. The best results follow its use in the morning before breakfast, the patient taking it while in bed, and remaining on the left side for ten minutes before rising. It may be taken oftener, but once a day may suffice, and it is advantageously used in this manner after the practice of lavage.

The hydrozone may at first produce acrid sensations in the stomach, but as the irritated gastric surface improves in tone under its influence, this will pass away and sensitiveness to its action will subside. Where necessary the amount of hydrozone may be reduced until the stomach becomes more tolerant to it.

The important step in chronic gastric catarrh, as in catarrh of all other mucous cavities, is the cleansing of the part from the ropy mucus, which clogs the glandular organs, and serves as a nidus for the operation of agents of fermentation. Glycozone in teaspoonful doses, diluted with water, administered after meals, prevents fermentation of food and accelerates a cure.

If the treatment outlined above be properly carried out the writer believes that little more is necessary, for with the removal of morbid accumulations the gastric secretions will become normal in quantity and quality. Hydrochloric acid, administered internally, may in some cases do good, as also the bitter tonics, but their place is secondary to the use of the stomach tube and the disinfection of the mucous membrane of the stomach with hydrozone.—*New England Medical Monthly.*

---

### THE ACTION OF SCOPOLAMINE UPON THE IRIS AND CILIARY MUSCLE.

Dr. Chas. A. Oliver, in the *American Journal of Medical Science*, reports the following observations and conclusions upon scopolamine:

*Observations.* 1. The mydriasis of a single instillation of the one-four-hundred-and-eightieth of a grain of hydrobromate of scopolamine is obtained in eighteen minutes.

2. The ciliary paralysis of a single instillation of the one-four-hundred-and-eightieth of a grain of hydrobromate of scopolamine is consummated in twenty-three minutes.

3. The single instillation of the one-four-hundred-and-eightieth of a grain of hydrobromate of scopolamine produces full dilatation of the pupil in every instance.

4. The dilatation of the pupil occasioned by the single instillation of the one-four-hundred-and-eightieth of a grain of hydrobromate of scopolamine remains *ad maximum* from twenty-four to thirty hours.

5. The total ciliary paralysis produced by the single instillation of the one-four-hundred-and-eightieth of a grain of hydrobromate of scopolamine is maintained from twenty-four to thirty-six hours.

6. By accurate observations made several times daily after the mydriasis and ciliary paralysis of the single instillation of the one-four-hundred-and-eightieth of a grain of hydrobromate of scopolamine are obtained, it is found that the diameter of the pupil becomes normal in about seventy-two hours, and total re-establishment of the power of the ciliary muscle takes place in about ninety-six hours' time.

During the course of experiments with the drug it was noticed:

1. At the beginning of a few of the examinations there was a slight sense of conjunctival astringency which in a couple of instances amounted to a stinging sensation.

2. There were no appearances of constitutional disturbance; care, however, having been taken in every instance to prevent the passage of any of the liquid into the lachrymal passages.

3. In no instance was any apparent increase of a choroidal disturbance produced by the employment of the drug used.

*Conclusions.* 1. The early and complete paralysis of the ciliary muscle that can be obtained by the single instillation of the one-four-hundred-and-eightieth of a grain of hydrobromate of scopolamine and the rapid and full return of the action of the muscle render this drug in this amount the most efficient and the most valuable cycloplegic that can be used for the proper determination of the total amount of ametropia.

2. The comparatively rapid return of the full dilatation of the pupil produced by the single instillation of the one-four-hundred-and-eightieth of a grain of hydrobromate of scopolamine to normal pupillary width renders the drug in this strength less objectionable than those drugs which by reason of necessarily greater strengths, to afford proper cycloplegic work, must be employed in amounts that give more permanent mydriasis.

3. The perfect freedom from injurious constitutional effects when the one-four-hundred-and-eightieth of a grain of hydrobromate of scopolamine is used renders the drug in this amount absolutely safe for employment in all cases in which total cycloplegia becomes necessary.

---

### ANTISEPTIC AND CLIMATIC TREATMENT OF PULMONARY TUBERCULOSIS.

Concluding an address before a late meeting of the American Climatological Association, Dr. E. Fletcher Ingals, of Chicago, said (*Jour. Am. Med. Association*):

As I have elsewhere stated, I believe that, all told, about 33 per cent. of patients with pulmonary tuberculosis recover under ordinary conditions, and I think that patients sent early to a high altitude and dry atmosphere have their chances increased fully 50 per cent. This belief is based upon the well-known fact that the records of autopsies show that in 25 per cent. of bodies dying from other diseases than pulmonary tuberculosis, the previous existence of this disease is demonstrated in the apices of the lungs; also upon the oft-repeated statement that a large percentage of those sent early to a good climate recover; and further, upon my personal observation of many cases. After consolidation of the apex of the lung has extended below the third rib, by which time the second stage is generally fully established, I feel that I have ample reason for believing that from 15 to 30 per cent. may be greatly benefited by climate, although life is seldom prolonged more than five or six years. Even after breaking down of the lung tissue has begun, a few may have their lives considerably prolonged by suitable climate. I have no doubt, however, that in the latter part of the disease the fatal result is generally hastened by the fatigue and the mental and physical distress incident to the journey. The physician should carefully study not only the physical, but the social and financial conditions of his patient before recommending a change, and while it is not necessary to say it to the members of this Association, I wish to impress upon others that it is neither scientific nor kind to send patients with consumption indiscriminately to places from which we have simply heard favorable reports. As a rule, in the first stage a warm climate is most salutary, but it is not so important, providing an abundance of sunshine and a dry atmosphere are obtained, although many phthisical patients are better in a southern latitude in winter. It will be found that patients who feel best in winter are likely to be benefited by a comparatively cool climate, the others in a warmer temperature. In the first stages it is desirable, when there are no contraindications, that patients go to an altitude of from six to seven thousand feet, but this is not suitable for persons who are nervous to a marked degree or who have a high temperature, or who have pronounced cardiac disease, emphysema, or laryngeal complications. Laryngeal tuberculosis is generally markedly aggravated by high altitudes. Hemoptysis is not, as is often supposed, a contraindication to a sojourn in a high altitude. On the contrary, bleeding is often promptly checked by this change, and those who seldom or never have hemorrhages in a high altitude frequently experience them quickly upon return to a lower level. In the second stage of the disease a high altitude is often beneficial, but we can not feel so certain of its results, therefore it is best to send the patient to an altitude of not more than two or three thousand feet, and if they do well subsequently advise the higher level. A warm, moist atmosphere seldom seems to have any useful effect in prolonging the life of consumptive patients, though it undoubtedly adds to their comfort in certain instances by relieving the irritability of the bronchial mucous membrane. The majority of patients who are unable to take care of themselves seldom receive much benefit from a change of climate, but as a few of them do, the experiment is constantly made, more often, I think, by the friends than by the physician. Wherever practicable, even in the best cases, it would be desirable for friends to accompany the invalid, because we must not forget the necessity of maintaining the nutrition, not only by suitable food, but by cheerful environment foreign to those who, sick and lonesome, find themselves the prey of nostalgia. Before recommending a patient to change climate, we should inquire as carefully as practicable as to his disposition and social and financial condition. If he is unable to obtain the comforts and some of the luxuries of life away from home, it is generally unkind to recommend any change of climate, because the chances are greatly that it would do more harm than good. If he is despondent he should have a cheerful companion. If he is in constant terror from the bacilli floating in the atmosphere, or is depressed by the sight of sickness in others, he should be removed as far as possible from other invalids suffering from tuberculosis. Indeed, this would be a good rule in many cases, but it is hardly ever practicable, because as soon as a locality obtains a reputation in the cure of tuberculosis, many invalids are attracted thither. The physician must remember that he has a duty to others as well as to the patient, and that he must sometimes consider the healthy friends, or more particularly the widow and helpless children soon to be thrown upon the world. Hard-hearted as it may appear, the physician must then answer to his conscience whether it is right to advise the friends to make great sacrifices with the hope of prolonging the invalid's life for a few weeks or months. As a general rule, our duty is to the patient only, but this, like other general rules, is subject to many exceptions. Unhappily, it is only in the minority of cases that experts in the Eastern or Middle States are consulted as to the wisdom of a change of climate by the patients who come under their observation. The great number come with their minds already made up to go somewhere, so that we can only guide them to the least objectionable place. This explains the fact often observed at health resorts, that the majority of patients sent away might better have been kept at home.

In conclusion, in addition to tonic, supporting and anodyne remedies, various antiseptics appear to possess great value in the treatment of pulmonary tuberculosis, but in order to get good effects it is imperative that the system be as nearly saturated with them as possible. They should be given at first in small doses, but the amount should be steadily increased until the maximum dose is obtained, care being always taken not to disturb the digestive organs. For example, with the oil of cloves we may begin with five drops to be given in capsule from three to five times daily after each meal and in the middle of the forenoon and of the afternoon, the medicine always to be followed by a glass of milk. The second day the dose should be six drops, the third seven drops, and so on, until a dose of twenty-five or thirty drops is given each time.

Creasote can seldom be given in sufficient quantity to have any material effect, because of the disturbance of the digestive organs which it is liable to cause and because of its coagulating effect upon all albuminoids. The same may be said of carbolic acid.

Carbonate of creasote is much more bland and may be given in doses of from five to six drops each meal with great benefit.

Guaiacol may be given in much the same way as the oil of cloves, though in somewhat smaller doses, but it is usually less easily borne than the carbonate of creasote or oil of cloves and often can not be tolerated in sufficient quantity. The carbonate of guaiacol may be used in much the same way as guaiacol itself, but most patients seem unable to take it in sufficient doses.

Oil of cloves and carbonate of creasote are the most satisfactory antiseptics for internal use. Iodin may be used as recommended by Shurly with undoubted benefit, but it causes considerable pain and is open to the objection that it necessitates too constant attendance of the physician; it may also be used advantageously as an inhalent.

Patients should not be sent from home unless their financial and social condition is such as to render the journey and sojourn easy and agreeable.

In the first stage of the disease patients should as a rule go to a high altitude where the atmosphere is dry and as warm as practicable. In the second stage they should be sent to a medium altitude and in the advanced stage, if sent anywhere, it should be to a low altitude.

Patients who have been improving on any course of medication should not discontinue it upon going to a different climate, but, however valuable any remedy may appear, it should not be continued if it becomes clear that it is deranging the digestion.

When sojourning in a favorable climate the patient should be out of doors as much as practicable during the pleasant portion of the day, should avoid excessive heat, excessive cold and unusual fatigue.

Of anodynes to check cough hyoscyamus, camphor, cannabis indica, stramonium and conium are of the most value, because they can generally be taken in sufficient quantities without disturbing the digestion, whereas opiates are usually deleterious in whatever form they may be employed.

The majority of patients sent from home in the latter stages of pulmonary tuberculosis are injured by the journey and their lives correspondingly shortened, though in a small percentage very great benefit is obtained in a warm and very dry climate.

---

### APENTA BITTER WATER.

W. S. Bogoslowsky, from clinical observations on the action and value of a constant bitter water, draws the following conclusions (*Transactions* of the Moscow Section of the Society for the Preservation of Public Health, No. VI.): Systematic treatment with Apenta water is especially indicated for constipation produced by atony of the bowels, and it has the advantage that its use does not give rise to subsequent constipation. Its action is more gentle than that of some other bitter waters because it contains less calcium sulphate and no magnesium chloride. It is probably owing to this circumstance that it does not cause crampy pains. The efficiency of Apenta as a remedy for the systematic treatment of obesity is clinically established.—*Brit. Med. Journ., August 25, 1897.*

---

### SOUTHERN STATES HEALTH RECORD.

*Florida Health Notes*, March, 1897, gives some interesting statistics, which "show up" the Southern States in a way which we had not looked at before. When the United States census was taken, the question was asked by the census-takers of the heads of each family in the United States, how many members of the family had died during the twelve months ending with June. The enumerators did not depend upon reports of physicians or health officers, but got their information direct from the people, and it was as likely to be correct in the South as in the North—in Florida as in Massachusetts. The census of 1890 also shows that the death-rate in the Southern States was lower than in the Northern States—much lower than in the New England and other North Atlantic States—notwithstanding the death-rate of the South includes the count of negroes, whose mortality is proverbially equal to, if not greater, than the birth-rate.

The following table tells the story, giving the deaths per 1,000 inhabitants in each of the States west of the Mississippi river during the year ending with June, 1890. For the purpose of comparison, we place the Northern States in a column to the left, while the Southern States are placed in the right-hand column:

| NORTH. | | SOUTH. | |
|---|---|---|---|
| Rhode Island | 21.9 | Delaware | 18.4 |
| New Jersey | 21.0 | Maryland | 17.3 |
| New York | 20.5 | Virginia | 14.0 |
| Massachusetts | 20.1 | Alabama | 13.0 |
| Connecticut | 19.4 | South Carolina | 13.5 |
| New Hampshire | 18.8 | Tennessee | 12.9 |
| Vermont | 16.3 | Kentucky | 12.0 |
| Maine | 15.8 | Georgia | 11.0 |
| Pennsylvania | 14.0 | Mississippi | 11.5 |
| Illinois | 13.9 | North Carolina | 11.4 |
| Ohio | 13.6 | West Virginia | 10.9 |
| Michigan | 11.9 | Florida | 10.1 |
| Wisconsin | 11.1 | | |
| Indiana | 11.0 | | |
| Average North | 15.8 | Average South | 13.0 |

The States west of the Mississippi, or the majority of them, are of so recent settlement, that a comparison with them would be misleading. They were settled by the young and vigorous, and until they are older, their death-rate will be small, whether the conditions which surround the people are healthful or the reverse.

Florida's death-rate is lower than that of any of these States, in spite of the fact that so many sick people go there, and that many of them die there. The death-rate of California, also a health resort, and a younger State than Florida, was 14.7 per 1,000 in 1890, and it was increased by the same cause that increased the death-rate of Florida.

The census of 1880 also shows Florida with the lowest death-rate of any State east of the Mississippi, and in 1880, as in 1890, the death-rate in West Virginia was next lowest. The fact that Florida held this enviable record for two decades in succession shows that the conditions for health that surround the people of Florida, summer as well as winter, are the very best.—*Virginia Medical Semi-Monthly.*

---

### PROBABLY THE FIRST CESAREAN SECTION IN GEORGIA.

The records of the Catholic church, of Savannah, contain the following description of what was probably the first Cesarean operation in Georgia (*Ga. Jour. of Med. and Surg., Savannah*):

On Wednesday, the twelfth of March, one thousand eight hundred and six, was buried in the graveyard of Savannah (after being carried to the Roman Catholic Church of said city, where common prayers were performed by the assistants, there not being a regular clergyman at present), the body of N. N. Pouyat, lawful daughter of John Francis Pouyat, residing in this city, and of Maria La Maignere, deceased, being extracted alive, by surgical operation, from the womb of the corpse of her mother, on yesterday, the eleventh instant, and dead at about six o'clock in the afternoon on the same day, after having previously received the baptismal water, administered by a friend present, in want of a clergyman. The said surgical operation being performed by John Baptist Berthelot and Nicholas Nazaret, physicians and surgeons of this city, and in the presence of James Phillips Rossignol de Grandmont and Picot de Closrivieres, who, said physicians and witnesses, have been requested by Anthony James DuFaure La Jarte, cousin of the deceased, and in the name of the other parents and relatives, to make their declaration respecting the position, time and circumstances of the death of said deceased infant, as above related, to be inserted into the present, on account of the difference of the form and laws of the United States, and for the future interests and civil concerns of the family as the case may require. Were also present at said funerals, a number of relations and friends, part of whom have likewise signed the present with me, as Secretary to the Vestry and depository of the records of this church. (Signed)

N. Nazaret, P. Berthelot, Lewis Grand Dutreuith, E. Rossignol, Lewis Rossignol, J. P. Rossignol Grandmont, Picot de Closrivieres, Dufaure La Jarte Montrelet.

THOMAS DUPENAUN,
Secretary.

---

### A STUDY OF PYEMIA AND SEPSIS.

Hentschel, in a monograph (*Cent. fur Chir.*, 1896, No. 40), in studying this form of disease, classifies its manifestations under the forms of pyemia, septicemia, sepsis, and sapremia.

Pyemia is that condition produced solely by pyogenic micro-organisms. Septicemia is that form in which the micro-organisms develop in the blood only (rare in man). Sepsis and sapremia are systemic intoxications arising from a local infection.

He illustrates pyemia by a case in which a tendo-vaginitis of the index-finger was followed by a suppurative arthritis of the shoulder-joint. The diplococcus lanceolatus (Fränkel-Weichselbaum) was found by microscopic investigation and cultures at both points, but would not produce suppuration after inoculation into animals.

In a second case pyemia followed a furunculosis of the lip. The culture showed staphylococcus citreus and other forms.

Two other cases illustrate typical sepsis—*i. e.*, toxemia. In both cases there was an absorption of toxins after gangrenous processes. One was a compound fracture of the leg followed by consecutive sepsis, which became systemic despite an amputation. The bacteriological examination showed the blood to be entirely free from bacteria or spores.

The second case on which a post-mortem examination was made showed how extensive the systemic involvement was. A periproctal abscess, with no tendency to healing, gave rise to the systemic infection. No bacteria were found in the blood. There was marked cell-necrosis. The symptoms were those of grave pyemia. The author is of the opinion that in the production of these cases of pyemia the absorption of the toxins is not the only question, but that the interchange between the living bacteria and the cells of the body is of more importance, although it should not be forgotten that the intoxication of saprophytic and pyogenic micro-organisms is greatly increased by their combination.—*Am. Jour. Med. Sci.*

---

### Specialists and Specialties.

A medical specialist differs widely from the poet, who, we are told, is "born, not made." The medical specialist is made, not born. No specialist has ever yet been born full-fledged from a medical college, though occasionally a young man imagines that such a miracle has been performed. The fledgling who supposes that a medical diploma and a post-graduate course can make a specialist, deceives himself and endangers the safety of the community. Such specialists have done untold harm to the profession. The eye, the ear, the skin are not simple appendages, each performing its duties independent of the rest, to be treated without reference to the other organs and the condition of the body at large. The successful specialist must also be a competent general physician.—*Ex.*

---

### GYNECOLOGICAL GLEANINGS.

In chronic ovaritis, pain is an inevitable feature, and nineteen times out of twenty it is worse on the left side than on the right. —*Tait.*

Give iron when the menses are scanty and lack color; give arsenic when the flow is too profuse, prolonged or frequent.—*Fordyce Barker.*

Cancer of the womb usually begins on the vaginal portion of the cervix, and consequently has to bear the brunt of the insults of coition and parturition.—*Goodell.*

The most common displacement of the ovary is dislocation downward into the retro-uterine pouch, to which the name of prolapse has been improperly given.—*Tait.*

Chronic leucorrhea of long standing can be cured only by persevering in frequent local use of astringents, through a speculum, together with hot vaginal injection.—*Munde.*

Tepid vaginal injections, so generally recommended and inadvertently used by patients in place of the hot injections directed, have no positive therapeutic effect whatever.—*Barnes.*

The ovary is simply a gland, developed as other glands and formed of similar elements; its peculiarity is that its cell-nuclei have special powers during a certain time of life.—*Tait.*

The peculiar sensation imparted to the finger on drawing a curette over the endometrium may give some hint as to the nature of the affection; if it is grating, it is vegetations or placental fragments; if soft and spongy, it indicates endometritis hyperplastica. —*Munde.*

